# It's the talk: a study of involvement initiatives in secure mental health settings

**DOI:** 10.1111/hex.12232

**Published:** 2014-07-07

**Authors:** Mick McKeown, Fiona Jones, Karen Wright, Helen Spandler, Joanna Wright, Holly Fletcher, Joy Duxbury, Jolene McVittie, Simon, Wayne Turton

**Affiliations:** ^1^ School of HealthUniversity of Central LancashirePrestonUK; ^2^ Community FuturesPrestonUK; ^3^ School of Social WorkUniversity of Central LancashirePrestonUK; ^4^ Secure & Specialist Mental Health Commissioning TeamYorkshire and HumberUK; ^5^ Garrow HouseYorkUK; ^6^ Involvement Strategy GroupYorkshire and Humber Secure ServiceUK

**Keywords:** communicative action, democracy, involvement, mental health, secure services

## Abstract

**Background:**

A study of involvement initiatives within secure mental health services across one UK region, where these have been organized to reflect alliances between staff and service users. There is little previous relevant international research, but constraints upon effective involvement have been noted.

**Objective:**

To explore and evaluate involvement initiatives in secure mental health settings.

**Design:**

A case study design with thematic analysis of qualitative interviews and focus groups.

**Setting and participants:**

Data collection was carried out between October 2011 and February 2012 with 139 staff and service users drawn from a variety of secure mental health settings.

**Findings:**

Our analysis offers four broad themes, titled: safety and security first?; bringing it all back home; it picks you up; it's the talk. The quality of dialogue between staff and services users was deemed of prime importance. Features of secure environments could constrain communication, and the best examples of empowerment took place in non‐secure settings.

**Discussion:**

Key aspects of communication and setting sustain involvement. These features are discussed with reference to Jurgen Habermas's work on communicative action and deliberative democracy.

**Conclusions:**

Involvement initiatives with service users resident in secure hospitals can be organized to good effect and the active role of commissioners is crucial. Positive outcomes are optimized when care is taken over the social space where involvement takes place and the process of involvement is appreciated by participants. Concerns over risk management are influential in staff support. This is germane to innovative thinking about practice and policy in this field.

## Introduction

This paper reports a study of involvement initiatives in secure mental health settings across the UK Yorkshire and Humber region, developed through alliance building between service users and staff.^1^, ^2^ Qualitative interviews and focus groups, nested within a broader case study design, elicited views of service users, staff and commissioners on the value and impact of approaches to involvement. Service user involvement was integral to all stages of the research process, with an ex‐user of secure services as research assistant, and current service users and staff forming a reference panel for planning and analysis. Findings indicate involvement practices are well developed in key aspects of secure services, overcoming previously noted limitations,^3^ but some require further development.

## Involvement practices in secure settings

Secure, or forensic, mental health services provide care for individuals who enter via courts or prisons or present significant management problems within non‐secure environments.^4^ UK services are configured at high, medium and low security levels with a concomitant focus on risk management. This study focuses only on medium and low secure units. Despite user involvement being an important part of UK policy,^5^, ^6^ and a tradition of survivor activism,^7^, ^8^, ^9^ there is sparse literature on involvement practices within secure services.^10^ Historically, service users in these settings were not routinely involved in the research process^11^, ^12^ although a cluster of projects have been funded to this end.^3^, ^13^, ^14^, ^15^, ^16^ Few studies report service user experiences in secure care,^17^ although some interesting first person accounts have been published.^18^, ^19^ A recent focus on recovery has initiated exploration of service users' accounts and involving them in inquiry.^20^, ^21^, ^22^

Different types of space or place might better support service user autonomy and empowerment.^23^ This is especially pertinent where security measures make a big difference to places people occupy and relationships therein. One UK study of user involvement in secure services^3^, ^10^ highlighted deficiencies in the quality of communication between service users and staff as characterized by lack of openness and honesty. The researchers experienced difficulties organizing the participatory project they initially envisaged because of limiting features of the secure environment. As an alternative, they brought a panel of people with experience of detention in secure care into the university setting, enabling more open dialogue.

Habermas's^24^, ^25^ communicative action theory was drawn on to make sense of this, and has been similarly deployed regarding involvement initiatives in mainstream mental health settings.^26^, ^27^, ^28^ For Habermas, change is driven by communication and forms of deliberative, participatory democracy. Ideally, participants should enter into these interactions with equal power, respect and open minds. Our findings resonate with these ideas, and reflections on communication and social space are taken up in the discussion.

In this particular region, co‐operative involvement networks, facilitated by Involvement Lead personnel, have supported a number of innovations. These utilize imaginative and creative approaches to participation, focusing on service user involvement in decision making, directing their own care and strategic‐ or policy‐level deliberations. The goals are individual empowerment, improved working alliances and smoother progress through the secure system. The Involvement Strategy Group, a region‐wide deliberative forum, uses experiential and socio‐dramatic techniques to support full participation and includes delegates from all secure units in the region. Work streams focused on particular developments are cascaded into similarly constituted Involvement for Innovation (i4i) meetings. These forums focus on strategic developments, and programmes like *My Shared Pathway* 29 have been taken up nationally. Standards for Care Programme Approach (CPA) have been developed that effectively place willing service users at the centre of organizing their own case reviews. A number of filming projects have trained service users to produce films publicising different involvement initiatives or assisting service users and families on transitions into secure units. Strategic involvement concerning Women's services sought solutions for some key problems facing women detained in secure units. A major outcome was the establishment of Garrow House step‐down facility, with concerted attention to the quality of the built environment and relational model of care. There are numerous other examples, including a joint staff‐service user football team in one of the medium secure units. More description of the various involvement initiatives can be found via the Commissioning Team website.^30^

## Method

The study comprised a series of case studies^31^ of particular involvement work. Participants were purposively recruited to reflect their involvement in different initiatives, allowing us to study these in depth. We restricted ourselves to a focus on four key examples: the ISG; the CPA standards; filming projects; and women's secure services. The research team met with the ISG before commencing the study, at regular intervals once underway and on completion. This contact was the springboard for recruitment, proceeding using snowballing techniques supported by key service user and staff champions for involvement in the various units.

In this paper, we restrict ourselves to reporting qualitative findings from semi‐structured interviews and focus groups. Data collection comprised of 60 individual interviews, 6 paired interviews and 10 focus groups comprising 67 participants. In total, there were 139 participants, comprising 70 staff and 69 service users, reflecting the extant alliance‐based approach to involvement, but also including people not engaged in involvement work. The different approaches to data collection were utilzed to potentiate unconstrained responses, with participants having choice of format. Table 1 provides more details about the participants. Paired interviews involved service users and staff who had formed close alliances in practice. Interviews lasted between 25 minutes and 2 hours, with the focus groups tending to last 2 hours. The data was subject to thematic analysis,^32^, ^33^ a process led by the research team in conjunction with the reference panel. The interviews and focus groups followed a fairly open topic guide, exploring participants' experiences of involvement practices, their impact, enabling or constraining factors, and how they made sense of this.

**Table 1:** Participants

	Medium secure	Low secure
Service users	29	40
Men	24	27
Women	5	13
Staff	39	26
Nursing	16	16
Health care assistants	4	4
Occupational therapy	8	3
Social work	5	2
Psychology	4	–
Psychiatry	2	–
Advocacy	–	1

Commissioning team (including Regional Involvement Leads): 5.

Ethical approval was granted by the Leeds Central Research Ethics Committee (11/YH/0315).

## Findings

Security features were felt to constrain full realization of involvement goals, and the most appreciated example of involvement was organized in a non‐secure, community setting. Nevertheless, experiences were complex and a range of involvement practices were possible within secure settings, delivering positive outcomes for engaged service users and staff. These issues were reflected in four themes spanning the different case studies which we summarize with illustrative quotes.

### Safety and security first?

This theme reflects tensions between involvement practices and concerns with risk management. For some staff, not necessarily against involvement in principle, the extent that involvement can be enacted uncritically in secure environments is limited, more so as one travels up security levels. This view was put strongly by one Charge Nurse in a medium secure unit:

On my ward it is safety and security first, involvement and anything else come second to this.

Other staff and service users argued that increasing levels of involvement and therapeutic alliance should promote shared knowledge of risk, improving effective management and, ideally, encouraging service users to become more responsible for their behaviour:

If you give people more choices it goes hand in hand with them becoming more able to exercise those choices responsibly … in the long‐run I think this makes people more responsible. Yes, I'd say if we promote autonomy, on the whole we minimise risk (Psychologist).

### Bringing it all back home

This theme focuses on challenges posed in extending involvement practices to all units at all levels. Staff and service users appreciated collective forms of involvement characterized by mutual respect, high quality communication and conducive setting; the centrally organized ISG exemplifying this. Involvement efforts that made a real difference to practice and policy were typically valued, as was the leadership role of the commissioning team. Similarly, co‐operation between service providers on target setting and quality improvement was appreciated. The presence of lead commissioners at the ISG indicated a meaningful level of influence:

You'll get general managers moaning to commissioners and you think yes, I'm part of something here … you get insights into the way things work… the whole [recites acronyms for commissioning targets] and such things (service user).

Sharing experiences with people from other units influenced demands for change:

like over the issue of mobile phones … it was great to hear what was happening on other units and the success they have had … it hasn't happened yet on our unit but it is good to know, and be able to say that other units allow this (service user).

The setting for the ISG was important – held in a non‐secure community venue with good facilities and making use of creative, participatory facilitation practices. Small groups of staff and service users often travelled to meetings together; continuing discussions and strengthening relationships.

Away from such central meetings, involvement practices at ward and unit level presented a mixed picture:

The community meetings here can be good, but mostly they are boring, and not everyone wants to go. They are definitely different from the bigger meetings [ISG]. Sometimes it is just the staff letting us know what is what (service user).

The importance of involvement practices being supported or not by staff or the extent to which different care systems or configuration of services facilitated involvement were noted. There were some perceived variations in the support of different professional disciplines for involvement. At its simplest, there was a contrast between ward staff, typically the nursing team, and other disciplines, usually based off‐ward, with the latter felt to be most supportive of involvement. Similarly, some psychiatrists were criticized for ‘old‐school’ attitudes towards involvement in clinical decision making concerning medication or leave.

Where trust was lacking, some service users resisted involvement. Often, in the period following first admission to a unit, they would find themselves fighting the system rather than co‐operating:

For years I kicked against it … I even went AWOL for a time. In those days, I wouldn't have got involved whoever asked. I didn't trust the staff and they certainly didn't trust me (service user).

### It picks you up

Involvement practices were recounted as having a positive impact upon well‐being and recovery yet also taxed people's emotions. Being part of meetings leading to tangible results afforded opportunities for positive affirmations of contributions, increasing confidence and self‐esteem:

I used to be nervous and would hang around in the background … now you can't stop me. I chaired my own CPA the other day and it was fantastic. Everyone let me know what a good job I had done… it felt a bit weird at first, but now I'd say everyone should be in charge of their own meetings (service user).

A growing culture of involvement at unit level could also assist service users in navigating the system; making the most of care‐team meetings or individual encounters with health professionals. Smaller numbers of service users found the amount of work they took on burdened their personal resources. Involvement practices also foregrounded service users' thinking about recovery and progress through the system:

If patients can give their view about what recovery is, you know, then that is crucial information for the care team, commissioners … and, ultimately, about letting people go: discharge from hospital (service user).

Participants' recollections were replete with the emotional characteristics and consequences of this work, mixing frustration and fulfilment. Tangible successes were related with pride, but there were challenges, sometimes attending boring meetings or becoming worn out trying to persuade people of an initiative's value. One service user expressed intense irritation that others could not grasp the importance of something he had worked hard on, contrasting this with good feelings when people were more positive:

It can feel like you are wasting your time … I had to listen to a lot of negativity during the pilot from other service users … you know, ‘what the fuck is this for’ …. It does get you down. I'm trying to help them you know … and then, some staff thought it was fucking brilliant … that picks you up (service user).

For service users, opportunities to engage in involvement activity, especially away from the secure environment, was an escape from oppressive features of secure care. There were also chances to meet peers from different units, and, indeed, alter relationships with care staff from one's own unit. Consequent changes in sense of self and identity had a positive emotional impact. Equally appreciated were opportunities for humour and enjoyment of the proceedings.

Staff committed to supporting involvement experienced job satisfaction, occasionally counteracting negative aspects of work in secure units or reconnecting with a wholesome self‐image. This speaks of alienating features of mental health care, and whether estrangement from a caring, progressive practitioner identity is most likely to occur when service users are subject to compulsion and incarceration. One staff participant reported feeling rescued from becoming burnt‐out in his role, countering a previously cynical outlook. Proselytisers for involvement reported relying on appreciative approaches, highlighting potential job fulfilment:

Find out what they do have a passion for … say to them ‘why did you come into the job? What do you enjoy when you come into work?’ you can get into a discussion and by the end of it … you have actually met, together (Involvement Lead).

### It's the talk

This theme stressed the importance of communication in advancing involvement work. One of the most interesting dimensions of participants' discourse was the valuing of mutual relations and the degree to which dialogue was associated with the process of change:

… by having conversations with people, persuading them, listening to what they have to say, and then coming back with more persuasion … you have to be convincing … you have to be prepared to listen, and think about objections, and reply with a better argument (social worker).

The value of each other's communication in the act of involvement was not necessarily dependent upon relative powers of expression; raw experiences and associated emotions would be just as influential as any neat turn of phrase. The sharing of stories featured in positive experiences of involvement, and participants appreciated the opportunity to tell us their stories. The importance of communicative acts was emphasized in the words of one interviewee who, after initially struggling to articulate his thoughts, eventually declared: *it's the talk*!

Many service users observed that one effect of participation in involvement initiatives was subsequently feeling more able to engage in constructive talk with care teams. Practitioners were appreciated if they were respectful of different opinions and took time to listen. Importantly, service users did not necessarily frame an ideal encounter in terms of having demands met; rather they valued the process by which concerns were attended to, placing most value on receiving meaningful explanations for care and treatment decisions:

You don't always get what you ask for. All I am after is a proper explanation and the chance to put my view across (service user).

Linking involvement efforts with change did not merely flow from self‐interest but a desire to benefit others:

I spent years moving through very slowly … the most important thing is getting out, or knowing you have a chance of getting out. I'm out now. I carry on with the involvement stuff to make a difference for those who are still in (service user).

This other‐regarding stance connected with a number of concerns about diversity, participation and access within involvement practices:

To steal a phrase, I feel we are all in this together … it is great to see the deaf guys and the guys with learning disability taking an active part … it must be hard for them. It is up to us to be patient … there is one bloke (name) who really struggles to get his words out clearly, but he is always willing, and the staff help him. I wish I had some of his energy (service user).

## Discussion

The importance of communication and relationships in underpinning effective involvement were crucial in this study. Similarly, previous research and commentary identifies the empowering potential of communication if organized and supported in particular ways.^3^, ^23^, ^24^, ^25^ Hence, we have chosen to frame discussion of our findings around these concerns. The theme *It's the talk* offers an account whereby meaningful communication ameliorates constraining features of secure settings. Conversely, the *safety and security first* mantra can be seen to close down talk, and perhaps explain the limited extent that the most appreciated forms of involvement could be *brought back home* into routine ward environments. This confirms a long‐standing critique: that users have more control at abstracted levels such as policy advice, than in negotiation of their own care. Though forms of ‘deep dialogue’ can be enacted, various constraining factors intrude distance into formal caring relationships, in the extreme casting service users as ‘other’.^34^, ^35^

Livingston et al.^19^ described recognizable involvement practices in Canadian secure units, such as attempts to share power and responsibility or emphasize relational aspects of care. However, one of the few previous studies of involvement in UK secure care highlighted limitations of communicative quality or empowerment,^3^ with similar deficiencies bemoaned in non‐secure services.^23^, ^24^, ^25^ Arguably, most involvement initiatives do not reach necessary thresholds or setting conditions for realizing the empowerment potential of communication. For Habermas, this should not be restricted by deficits in power, mutual respect or connections across difference.

Lewis^36^ similarly points out difficulties for service users to set off on an equal footing in any communication with professionals who always have higher status and power. Such considerations are even more salient in secure settings, in the extreme closing down potential for constructive communication or silencing service user voices. Deliberative democracy is not a panacea in this regard, but creative approaches to deliberation evidenced in this study can contribute to ways in which Habermas's theory has been taken forward by wider critique in a context of disability and marginalization.^37^, ^38^, ^39^

Not least of the ways service user voices are constrained can be assumptions associated with psychiatric diagnosis and detention; most obviously a denial of capacity for rational debate. This is important because Habermas theorizes social change in terms of *reasoned* and *reasonable* communication. Coleman^40^ charts the extent psychiatric survivor activists have ‘significantly contributed to a reconfiguring of the relationship between madness and rationality’, possessing ‘a rational capacity to speak credibly about their condition and their treatment and… on the science of psychiatry’ (p341).

Emphasis upon *the talk* suggests that involvement initiatives in the region have partially overcome previous critical objections about limited communication. To some extent, this is explicable in terms of achieving a necessary ‘critical mass’ of interested participants. Empowered communication was most evident in the ISG, less so regarding routine ward‐based practices, indicating that social space may be the most telling factor enabling positive or empowering communicative acts. Observations on alliances connect with Habermas's stress on the relational, with staff‐service user and peer‐to‐peer relationships central to participants' appreciation for involvement. Commentary on the pertaining characteristics of valued communication echoes Habermasian deliberation; suggested as an appropriate vehicle for advancing movement politics and service user involvement in other contexts^41^, ^42^. These forms of decision making are characterized by taking time over discussion, the calm use of persuasion and counter‐argument, respectful attention and openness to reach consensus or change one's standpoint.

Relational models of care and security were particularly appreciated, occasionally resembling the democratic features of therapeutic communities, which might better potentiate Habermasian requirements for unconstrained communication. These places offered thorough systems for staff supervision with attention to the emotional labour of their work. Consideration of different sorts of social space available in secure units or associated with particular involvement initiatives raises awareness of tensions or contradictions that can lead to the sort of creativity wherein new possibilities can be imagined or brought about.^43^ Involvement could have a key role in enabling this if innovative practices begin to highlight such contradictions and promote reflection upon them; deliberations around risk management may be an obvious starting point. Tensions between relational and physical models of security play into concerns about forms of social space and place, and perhaps explain why some wards have been slower in taking up involvement practices.

A more critical view of involvement might emphasize processes of pacification, whereby individuals adjust to the system without too much fuss.^44^, ^45^, ^46^, ^47^ A pragmatic position would acknowledge limitations of involvement but work hard to reinforce the empowerment potential for service users to become as active as they possibly can in decisions and practices about their care. A true innovation would be to better include those who ‘kick against’ the system, perhaps necessitating a degree of flexibility to accommodate unruly and dissenting voices.^36^

The emotional flavour of involvement practices demonstrates both positive rewards of success and hard work trying to make involvement a reality in secure services. Staff can gain fulfilment in their work and sustain a valued professional identity. Effective involvement maximizes co‐operation and reduces tension and conflict in caring relationships, which in turn reduces job stress. That patients could frame their involvement as not motivated by self‐interest, opens up possibilities for individuals to access a positive self‐hood in direct contradiction of some of the more negative stereotyping of this client group.^48^ Service users obtain numerous benefits from involvement initiatives, connecting with recovery and well‐being, maximizing agency within important contexts, not least clinical decision making. These identity issues appear to be intimately bound up with processes of democratizing communication.

## Conclusion

There is evidence of systematic innovation supporting different involvement initiatives across Yorkshire and Humber. The scale and quality of involvement at the ISG are commendable for mental health services, let alone secure services. Leadership and investment from the Commissioning Team has been an important feature of these successes. Linking co‐operative networking practices to the setting, and mutual achievement, of Commissioning targets, represents an important innovation in driving up quality. This co‐operation is contrary to some of the principles of competition underpinning government policy but more in tune with many participants' values.^49^

Our analysis provides insights into the different ways in which people understand and appreciate opportunities for involvement. The importance of communication for effective involvement in these accounts connects with previous criticisms of involvement in mental health care, where the quality of communication was seen as insufficiently developed or supported to make involvement meaningful. The ISG was most appreciated by participants in our study and the non‐secure setting for this mirrors Godin and colleagues' transposition of discussions about secure care away from secure environs into the university. The fact that participants in our study could also speak of respectful and authentic communication for involvement within secure settings is testimony to the complexity of their experiences and suggests that efforts to organize involvement systematically can make a positive impact upon the constraining effects of security practices and culture.

That involvement practices might appear to be part of wider systems of social control, or pacification of dissent, is worth acknowledging. Habermas's theories offer useful insights, and point to the important characteristics of relationships and social space that might support or impede progressive developments. Further attention is required to address the complexities of deliberative communication in mental health service contexts with particular regard to tensions between equality of voice and authority. Novel forums for communication must be able to accommodate different contributions, including the recalcitrant, whilst maintaining mutual respect. This is likely to be of relevance across mental health services, beyond the walls of secure units.

## Conflict of interest

None.

## Funding

The research study was funded by the Specialist Secure Services Commissioning Team, Yorkshire and Humber.
